# Association between anxiety, depression and quality of life in male and female German students during the COVID-19 pandemic

**DOI:** 10.1186/s12888-024-05611-8

**Published:** 2024-03-18

**Authors:** Emily Wilzer, Annalena Zeisel, Veit Roessner, Melanie Ring

**Affiliations:** Department of Child and Adolescent Psychiatry, Faculty of Medicine Carl Gustav Carus, TUD Dresden University of Technology, Dresden, Germany

**Keywords:** Depression, Anxiety, Quality of life, Gender, Student, COVID-19 pandemic

## Abstract

Studies show that three-quarters of mental disorders appear during young adulthood, which makes students a risk group. Especially people with anxiety and depression experience lower Quality of Life (QoL) compared to healthy persons. Furthermore, previous research found that there was a wide range of negative mental consequences triggered by the COVID-19 pandemic. This study aimed to examine the association between anxiety, depression and QoL in male and female students at the time of the COVID-19 pandemic. Methods: 297 German students (121 men, age span_men_: 18–41 years; 176 women, age span_women_: 18–52 years) filled in the following questionnaires: World Health Organization Quality of Life Brief Version, Hospital Anxiety and Depression Scale and the Symptom-Checklist-90-R. Men and women did not differ significantly in their physical, psychological, environmental and global QoL. Results: While women showed higher raw anxiety scores, groups did not differ in terms of their raw depression scores. Furthermore, we found main effects of anxiety and depression on the four QoL subscales. Students´ QoL was highest if they were not affected by anxiety and depression, independently of gender. Psychological and social QoL was worst if the students reported marginal and particularly clinically significant levels of anxiety and depression. Men experienced worse psychological and social QoL than women for clinically significant anxiety levels. Conclusions: Interventions should target especially the psychological and the social subscales of QoL, as these areas are most affected by anxiety and depression. Possible interventions could be psychoeducational programs or participation in sports because it offers an opportunity for social interaction and goal-directed activity.

**Supplementary Information:**

The online version contains supplementary material available at 10.1186/s12888-024-05611-8.

## Background

If somebody has to explain a student´s life, they would likely mention keywords such as sleeping out, partying, meeting friends and sometimes going to a lecture, but the reality is mostly very different. Stress, pressure to perform, deadlines, fear of failure and fear of the future are probably experienced by most students. That is why it is important to investigate relevant factors like the Quality of Life (QoL) and mental health of those students.

QoL often includes terms such as well-being, functioning, life satisfaction, functionality and interference, and refers to those aspects of life that make it particularly fulfilling and worth living [1, 2]. The World Health Organization defines QoL as an “[…] individual´s perception of their position in life in the context of the culture and value systems in which they live and in relation to their goals, expectations, standards and concerns.” [3]. Health-Related Quality of Life (HRQoL) deals with an individual´s physical, functional, social and emotional well-being [4].

In general, numerous studies have shown that psychiatric disorders, especially co-existing symptoms of depressive or anxiety disorders significantly reduce the QoL of people in Western countries [5–8].

According to the Diagnostic and Statistical Manual of Mental Disorders [9], fear is a reaction to a real or perceived imminent threat and is mostly associated with increased arousal. Whereas anxiety is the anticipation of future threats and is accompanied by increased muscle tension, caution, and avoidant behaviors. On the other hand, common to all depressive disorders is a sad or irritable mood or a sense of emptiness, accompanied by physical and cognitive changes that severely impair the individual´s ability to function [9].

Returning to QoL, Zhou et al. [8] found that there is a possibility that generalized anxiety disorder (a form of anxiety disorder) leads to diminished psychological QoL, given the fact that psychological QoL can be viewed as a composite measure of mental health. They used the World Health Organization Quality of Life Brief Version [10] to measure QoL. To measure symptoms of anxiety and depressive disorders, the authors used the Hospital Anxiety and Depression Scale [8, 11]. Furthermore, Orley et al. [12] stated that “[t]here is no doubt that a depressed mood is likely to affect thinking in a generally negative sense, leading to a tendency to express dissatisfaction with most aspects of life” [12]. In a study by Rapaport et al. [13], patients with major depressive disorder (severe impact on QoL in 63% of patients), chronic/double depressive disorder (severe impact on QoL in 85% of patients) or PTSD had the lowest QoL-Enjoyment and -Satisfaction scores compared to a healthy control group. Whereas 21% of patients with social phobia disorder and 20% of patients with panic disorders experienced a severe impact on their QoL scores. Similarly, the review of Hansson [14] showed that people with major depressive disorder had lower subjective QoL than healthy subjects.

In addition to mental disorders, there are other factors that might be of relevance regarding QoL. Ellert & Kurth [15] investigated HRQoL in 7988 German adults who filled in a health survey that measures each of the following eight health domains: physical functioning, role-physical, bodily pain, general health, vitality, social functioning, role-emotional, and mental health. Men achieved significantly higher scores in QoL than women, except in the domain of general health. These gender differences were evident in all age groups, albeit at different levels.

Regarding students, approximately 75% of mental disorders appear during young adulthood, where anxiety and depression are the most common mental illnesses [16]. Some studies have shown that especially during the first years of study, the QoL of students deteriorated and symptoms of anxiety and depressive disorders increased [16, 17], which could mean that students are prone to develop mental disorders during their academic education. The study by Freitas et al. [18] included 321 health students and found that there was a negative association between the quality of life and depression symptoms in all examined domains (physical and psychological well-being, social relations and environment), while anxiety symptoms showed a negative association in the environment domain, and stress symptoms had a negative association in the psychological domain. Furthermore, Alsubaie et al. [19] found differences between students with and without depressive symptoms considering the psychological and social subscale of the WHOQoL-BREF. There, participants without depressive symptoms reported higher QoL scores in those two subscales. Similar findings could be shown by Ghaedi et al. [20]. Here, QoL scores were significantly lower in persons with social phobia disorder (another form of anxiety disorder) than in a healthy control group. In a sample of 6.198 German students, about a quarter reported high levels of stress (25.3%) with more female students reporting high stress levels (29.2%) compared to male students (21.4%) [21]. Against these findings, Schmidt-Gürtler [21] did not find significant differences between male and female psychology students in their self-reported stress levels. Looking at means, there were tendencies that women experienced slightly more stress than men. Davis et al. [22] examined 68 undergraduate college students and found men having significantly higher HRQoL than women. Again, in this study, women reported significantly more anxiety, depressive symptoms and hopelessness than men.

The COVID-19 pandemic is of relevance for this study, particularly social withdrawal and isolation. At the beginning of the pandemic, no one could have imagined the enormous impact this virus would have not only on our physical health but especially on our mental health. That is why it is important to have a closer look not just at the physical consequences of COVID-19 but also at the social and psychological consequences of the pandemic. In the face of school and business closures and the obligation to stay at home, the world has become much more shambolic, unpredictable and unsafe, which creates “[…] a breeding ground for stress, anxiety, and isolation.” [23]. Talevi et al. [24] found that there was a wide range of negative mental consequences triggered by the COVID-19 pandemic outbreak, for example anxiety, depressive symptom, stress, insomnia, avoidance, compulsive behavior and others. During the COVID-19 pandemic, 21.3% of college students reported mild, 6.33% moderate and 0.6% severe levels of anxiety, depressive symptoms and stress [24]. The authors also found that, among others, female gender and young adulthood (ages 18–40 years) were associated with higher levels of psychological distress. Further, Leaune et al. [25] found a higher prevalence of poorer mental health among French students during the first stage of the COVID-19 pandemic compared to other phases of the pandemic. Moreover, students reported poorer mental health and mental HRQoL, when they were affected by exam-related stress, pandemic-related financial problems, social isolation due to the lockdown and COVID-19-like symptoms or were female.

Overall, especially symptoms of anxiety and depressive disorders seem to be related to poorer QoL [9, 12–14, 17]. Female gender and young age were associated with higher levels of psychological distress [24, 26]. Moreover, patients with depressive disorders had poorer QoL-Enjoyment and -Satisfaction than patients with anxiety disorders [13].

Following the literature reviewed above, the aim of this study was to compare scores of anxiety and depressive symptoms among male and female German students and to examine the association between these two mental health problems and the QoL of those students during the COVID-19 pandemic.

Students with anxiety and depressive symptoms were expected to have poorer QoL compared to students without. In addition, we hypothesized that students with depressive symptoms would have poorer QoL than students with anxiety disorders. Moreover, we expected women compared to men to have higher anxiety and depression scores and thus, a poorer QoL.

## Methods

### Participants

As part of a larger research project, a sample of 297 German students (121 men, mean age_men_: 23.6 ± 3.9 years, range_men_: 18–41 years; 176 women, mean age_women_: 23.3 ± 3.8 years, range_women_: 18–52 years; see Table 1) was recruited between October 2020 and May 2021. Most of the students had A-levels (67.9%) or a degree from a university (18.5%) and most of them studied construction and environment (20.4%) or psychology (18.8%). Students often aspired to get the bachelor`s or master`s degree, diploma or the university degree required for e.g. teaching profession. Considering the mental status of included participants, 15 out of 121 men (12.4%) and 23 out of 176 women (13.1%) reported an F-diagnosis. An overview is presented in Table 1. There were no differences between the groups in terms of chronological age, *t*(295) = 0.61, *p* =.54, *d* = 0.072, course of study, *U* = 9413, *p* =.09, *r* =.1, frequency of F-diagnoses, *U* = 10,608, *p* =.92, *r* =.01, or educational status, *U* = 9243.5, *p* =.012, *r* =.15. Students were recruited via the student email distribution list including students of Dresden University of Technology and of Universitätsklinikum Carl Gustav Carus Dresden. Overall, 490 students got in touch, 348 individuals were screened, 310 students fulfilled the inclusion criteria, six of these individuals did not report back. In total, 305 students received the link to the online survey, seven of them did not fill in the survey because of a lack of time or private reasons. The final sample of fully completed questionnaires included 297 students. Inclusion criteria were: being a student of at least 18 years, speaking German fluently and fully completing the questionnaires. Exclusion criteria were consuming cannabis more than five times a year or using any other illegal drugs within the past year. Inclusion and exclusion criteria as well as demographic data were determined through a prior screening questionnaire.

**Table 1:** Demographic data

Variable	Male (*N* = 121)		Female (*N* = 176)	
Age in years	*M*	*SD*	*M*	*SD*
	23.62	9.96	23.34	3.79
	N	%	N	%
Educational status				
A-Levels	98	81	122	69.3
Higher education entrance Qualification	2	1.7	2	1.1
Higher education institution degree	6	5	7	4
University degree	15	12.4	45	25.6
Course of study				
Psychology	11	9.1	50	28.4
Mathematics and Natural Sciences	13	10.7	18	10.2
Construction and Environment	38	31.4	28	15.9
Engineering	25	20.7	8	4.5
Humanities and Social Sciences	9	7.4	34	19.3
Medicine	6	5.0	26	14.8
Teaching	19	15.7	12	6.8
Aspired degree				
Bachelor´s degree	33	27.3	75	43.8
Diploma	44	36.2	16	9
Master´s degree	17	14.1	44	25
University degree required for e.g. teaching	26	21.6	38	21.6
Postgraduate studies	1	0.8	-	-
Promotion / PhD	-	-	1	0.6
F-diagnoses^a^				
F30– F39^b^	5	4.1	10	5.7
F20– F29^c^	3	2.5	1	0.6
F40– F48^d^	5	4.1	7	4.0
F50– F59^e^	1	0.8	8	4.5
F60– F69^f^	2	1.7	0	0
F90– F98^g^	3	2.	1	0.6

*Note*^a^ Lifetime prevalence based on medical and self-reports. ^b^ Mood (affective) disorders. ^c^ Schizophrenia, schizotypal, delusional, and other non-mood psychotic disorders. ^d^ Anxiety, dissociative, stress-related, somatoform and other nonpsychotic mental disorders. ^e^ Behavioral syndromes associated with physiological disturbances and physical factors. ^f^ Disorders of adult personality and behavior. ^g^ Behavioral and emotional disorders with onset usually occurring in childhood and adolescence

### Procedure

Participants gave written informed consent prior to participation in the study. They answered the questionnaires online via LimeSurvey [27], which they accessed using a participant code. All questionnaires were German versions. It took participants approximately 60 min to complete all twelve questionnaires, three of which were of relevance for this project. Participants were compensated for their time with 10€ or Psychology students were offered 1 course credit upon completion. This study was approved by the ethics committee of the TUD Dresden University of Technology (Processing number: EK 356,092,018) and procedures adhered to the guidelines of Helsinki.

### Measures

To measure QoL, the 26 items of the WHOQoL-BREF [10] were used including the dimensions physical and psychological well-being, social relationships and environment. Participants answered the items on a 5-point scale. Subscale totals were transformed to a scale ranging from 0 to 100 with higher scores indicating higher QoL [3]. Internal consistencies for the subscales of the WHOQOL-BREF ranged from α = 0.57 to α = 0.88. We used total scores from the global QoL Item, the global item of overall health as well as from the four subscales for physical, psychological, social and environmental QoL.

Furthermore, we applied the Symptom-Checklist 90 Items Revised (SCL-90-R) [28], a questionnaire for the assessment of subjective impairment due to physical and especially psychological symptoms. The SCL-90-R consists of 90 Items assigned to nine symptom scales (somatization, obsessiveness, insecurity in social contact, depressiveness, anxiety, aggressiveness/hostility, phobic anxiety, paranoid thinking, psychoticism). Internal consistencies for the scales of the SCL-90-R ranged from α = 0.76 to α = 0.92, for the global characteristic score from α = 0.97 (adults). We measured perceived psychological distress using the Global Severity Index (GSI) [28] because the current study took place during the COVID-19 pandemic.

Finally, we used the German Version of the Hospital Anxiety and Depression Scale (HADS) [11] with 14 items to measure anxiety and depression. Participants rated seven items for anxiety and depression each on a 4-point scale. Subscale total scores can range between 0 and 21. Higher scores indicate more severe levels of anxiety or depression. In addition, scores were grouped into the following levels according to the cut-off scores: normal (0–7), marginal (8–10), clinically significant (11–21) levels of anxiety or depression [11]. The Cronbach’s alpha and split-half reliabilities for both subscales are 0.8 each.

### Data analysis

IBM SPSS Statistics [29] was used for statistical analyses. There were no missing data. Independent samples *t*-tests and Mann-Whitney U tests (for categorical data) were used for testing between-group differences. By using Multivariate Analyses of Variance (MANOVA) and follow-up Univariate Analyses of Variance (ANOVA), we analyzed the impact of group and levels of anxious and depressive symptoms on the QoL subscales of the WHOQoL-BREF. Furthermore, we used Pearson correlations to analyze the difference in men and women considering the possible connection between QoL and anxiety and depression. Significant results from MANOVA were analyzed by Scheffé’s post hoc tests due to uneven distribution of participants between groups. Cohen’s *d*, Pearson’s correlation coefficient *r* and partial eta squared (*η*_*p*_*²*) were used as measures of effect size.

All significance levels were set at 0.05 and Bonferroni correction was applied in the case of multiple comparisons.

## Results

### Group comparison of QoL, psychological distress, anxiety and depression for men and women

The descriptive data for QoL, psychological distress, anxiety and depression separated by gender are presented in Table 2. First, QoL (four subscales and global items) was compared between groups (men and women) without including anxiety and depression. Independent samples t-tests showed no differences between male and female students in the global QoL Item, *t*(295) = 0.96, *p* =.34, Cohen`s *d* = 0.11, the global item of overall health, *t*(295) = 0.28, *p* =.71, Cohen`s *d* = 0.04 and three of the four subscales of the WHOQoL-BREF, *t*_max_ ≤ 0.95, *p*_min_ ≥ 0.34, Cohen`s *d*_max_ ≤ 0.26. However, female students reported higher QoL in the social relationships subscale compared to male students, *t*(295) = 2.13, *p* =.03, Cohen`s *d* = 0.26. The distribution of the five possible answer categories (very good/satisfied to very poor/dissatisfied) for the WHOQoL-BREF global QoL item and the global item of overall health did not differ between male and female participants, *U*(global QoL) = 10058.00, *p* =.35, *r* =.05; *U*(global health satisfaction) = 10275.5, *p* =.56, *r* =.03. Moreover, the majority of male (65.3%) and female (60.2%) students rated their overall QoL as *good*. Some also rated their overall QoL as *neither poor nor good* (male: 19%, female: 18,2%). Similarly, most of the male (52.1%) and female (52.3%) students were *satisfied* with their overall health. Male students were second most likely to answer *very satisfied* (19.8%), whereas female students were most likely to answer *neither satisfied nor dissatisfied* (21.0%; see Table A1 in the Additional Files).

**Table 2:** Descriptive statistics for World Health Organization Quality of Life Brief Version (WHOQoL-BREF), Symptom-Checklist-90-R (SCL-90-R) and the Hospital Anxiety and Depression Scale (HADS) separated by gender

	Male		Female			Male		Female	
	M	SD	M	SD		n	(%)	n	(%)
**WHOQoL-BREF** ^**a**^									
Global QoL	70.87	17.48	72.87	17.90					
Overall health	69.63	23.54	68.61	22.64					
Physical well-being	78.93	13.33	77.86	11.89					
Psychological well-being	65.39	15.75	63.64	15.63					
Social relationships	64.39	20.89	69.41	18.55					
Environment QoL	73.27	11.66	74.08	11.10					
**SCL-90-R** ^**b**^									
GSI^c^	0.44	0.45	0.51	0.51					
PST^d^	25.11	16.66	28.87	17.34					
PSDI^e^	1.38	0.42	1.44	0.44					
**HADS** ^**f**^									
Depression^g^	4.73	3.60	4.26	3.05					
Normal	3.29	2.23	3.30	2.04		96	(79.3)	150	(85.2)
Marginal	8.67	0.72	8.83	0.86		15	(12.4)	18	(14.9)
Clinically significant	12.60	1.65	11.88	1.36		10	(8.3)	8	(4.5)
Anxiety^g^	5.99	3.59	6.87	3.62					
Normal	4.49	1.83	4.63	1.89		93	(76.9)	111	(63.1)
Marginal	8.78	0.88	8.95	0.84		18	(14.9)	38	(21.6)
Clinically significant	14.90	3.07	13.15	1.81		10	(8.3)	27	(15.3)

*Note*^a^World Health Organization Quality of Life Brief Version ^b^Symptom-Checklist-90-Items Revised ^c^Global Severity Index ^d^Positive Symptom Total ^e^Positive Symptom Distress Index. ^f^Hospital Anxiety and Depression Scale ^g^Hospital Anxiety and Depression Scale - Anxiety and Depression scores interpreted (levels according to cut-off scores). Values are rounded

Considering the overall burden of included participants, assessed with the SCL-90-R, there were no between-group differences in impairment in general (GSI), *t*(295) = 1.41, *p* =.16, Cohen`s *d* = 0.17, and in the intensity of impairment (PSDI), *t*(295) = 1,28, *p* =.2, Cohen`s *d* = 0.15. However, female students showed a tendency to report more symptoms (PST) compared to male students, *t*(295) = 1,87, *p* =.06, Cohen`s *d* = − 0.22, (see Table 2).

An independent samples t-test showed higher HADS anxiety raw scores for female compared to male students, *t*(295) = 2.06, *p* =.04, Cohen`s *d* = 0.24. No between-group difference was found for HADS depression raw scores, *t*(222.18) = 1.18, *p* =.24, Cohen`s *d* = 0.14. In addition, the distribution of levels according to the cut-off scores (see Table 2 for numbers and percentages) differed between the two groups in the anxiety subscales, *U* = 9126.5, *p* =.01, *r* =.15, with more women (16.5%) compared to men (6.6%) reporting clinically significant levels of anxiety. However, groups did not differ in the distribution of depression levels, *U* = 9991, *p* =.17, *r* =.08.

Further, to investigate the associations between variables, Pearson correlation analyses were run. The data are presented in Table A2 in the Additional Files. There were negative correlations between global QoL and HADS depression scores which were different for men (*r* = −.47, *p* <.001, *Z* = − 0.51) and women (*r* = −.40, *p* <.001, *Z* = − 0.42). Interestingly, men showed stronger correlations between global QoL and HADS anxiety scores (*r* = −.38, *p* <.001, *Z* = − 0.40), indicating lower global QoL for men with higher anxiety scores compared to women (*r* = −.22, *p* <.001, *Z* = − 0.22).

### Combined impact of Group, anxiety and depression on QoL

A three-way MANOVA was calculated by entering Group (male, female), Anxiety (normal, marginal, clinically significant) and Depression (normal, marginal, clinically significant) as independent variables and the four subscales of the WHOQoL-BREF (physical and psychological well-being, social relationships, environment) as dependent variables. There was no effect of Group on the dependent variables, Pillai´s trace = 0.06, *F* (4, 276) = 0.40, *p* =.81, ŋ_p_^2^ = 0.006. There were, however, effects for Anxiety on dependent variables, Pillai´s trace = 0.118, *F*(8, 554) = 4.347, *p* <.001, ŋ_p_^2^ = 0.059, as well as Depression, Pillai´s trace = 0.172, *F*(8, 554) = 6.535, *p* <.001, ŋ_p_^2^ = 0.086.

Follow-up ANOVAs revealed main effects of Anxiety for three WHOQoL-BREF subscales [physical, *F*(2, 288) = 17.24, *p* <.001, ŋ_p_^2^ = 0.107, psychological, *F* (2, 288) = 13.1, *p* <.001, ŋ_p_^2^ = 0.083, and environment QoL, *F*(2, 288) = 3.28, *p* <.04, ŋ_p_^2^ = 0.02] but not for social relationships [*F*(2, 288) = 0.055, *p* =.95, ŋ_p_^2^ = 0.00]. Bonferroni posthoc tests showed differences in QoL between the anxiety levels normal, marginal and clinically significant (*p*_max_ < 0.001). As can be seen in Table 3, the highest QoL was found at normal levels of anxiety and QoL was lowest at marginal and clinically significant levels of Anxiety for psychological and social QoL.

**Table 3:** Means and standard deviations of WHOQoL-BREF Quality of Life subscales by Group and level of Depression/ Anxiety

	Depression					Anxiety						
	Normal		Marginal		Clinically significant		Normal		Marginal		Clinically significant	
Measures	*M*	*SD*	*M*	*SD*	*M*	*SD*	*M*	*SD*	*M*	*SD*	*M*	*SD*
Physical well-being male female	81.40 80.05	10.67 10.56	75.95 67.66	11.01 12.52	59.64 59.81	22.08 5.96	82.03 81.85	9.66 9.61	76.59 72.46	12.39 12.45	54.29 69.05	18.50 12.45
Psychological well-being male female	69.75 66.92	12.76 13.41	53.50 48.15	14.07 15.21	42.92 36.98	15.75 7.85	69.13 69.44	13.17 12.45	59.03 56.47	17.63 14.06	42.08 49.85	10.84 13.50
Social relationships male female	67.71 72.11	20.06 17.30	57.22 58.33	17.50 18.30	43.33 43.75	19.95 13.91	67.39 71.17	20.10 18.80	58.79 66.45	19.49 17.38	46.67 66.39	21.59 18.85
Environment QoL male female	75.07 75.33	11.33 10.40	68.75 68.11	10.02 11.57	62.81 62.11	10.57 13.62	75.30 76.97	10.79 10.57	69.62 69.74	9.21 11.26	60.94 68.29	14.97 8.83

*Note* Values rounded. The higher values indicate a higher level of quality of life (QoL)

Similarly, there were main effects of Depression on all four WHOQoL-BREF subscales [physical, *F*(2, 288) = 16.89, *p* <.001, ŋ_p_^2^ = 0.11; psychological, *F*(2, 288) = 31.43, *p* <.001, ŋ_p_^2^ = 0.18; social relationships, *F*(2, 288) = 14.98, *p* <.001, ŋ_p_^2^ = 0.09, and environment, *F*(2, 288) = 6.87, *p* =.001, ŋ_p_^2^ = 0.05]. Bonferroni posthoc tests showed differences in QoL between all three Depression levels (*p*_max_ < 0.001). The highest QoL was found at normal levels of Depression. QoL was lowest at marginal and clinically significant levels of Depression for psychological and social QoL (see Table 3).

Additionally, there was an interaction between Group and Anxiety, *F*(8, 554) = 2.28; *p* =.02, ŋ_p_^2^ = 0.03, with women reporting higher anxiety scores than men. Bonferroni posthoc test showed differences in all subscales between the normal, marginal and clinically significant levels, except for normal and marginal and marginal and clinically significant in the social subscale and except for marginal and clinically significant in the environmental subscale. However, there were no interactions between Group and Depression, *F*(8, 554) = 0.28; *p* =.97, ŋ_p_^2^ = 0.004, and between Anxiety and Depression, *F*(16, 1116) = 0.86; *p* =.61, ŋ_p_^2^ = 0.012.

## Discussion

The aim of this study was to investigate the association between anxiety, depression and QoL according to self-reports in male and female students during the COVID-19 pandemic. We expected students with symptoms of anxiety and depression to report poorer QoL compared to students without symptoms of anxiety and depression. Secondly, we hypothesized that participants with depressive symptoms have poorer QoL than students with anxiety symptoms. Lastly, we expected women compared to men to report higher anxiety and depression scores and, therefore, a poorer QoL.

Against our Hypothesis 1 and past research [22, 24, 26], we did not find any differences between male and female participants considering the global QoL and three of the four QoL subscales (psychological, physical, environmental) of the WHOQoL-BREF. This is in line with Schmidt-Gürtler [21], who could not find any differences between women and men in global QoL and three of the four subscales (psychological, physical, environment) of the WHOQoL-BREF. A possible explanation for this could be a small sample size or because men and women were generally both burdened by the COVID-19 pandemic. Similarly, the author did not find any differences between male and female persons in experiencing stress, which the author explained by errors due to the sampling bias. According to the mean scores, female students tended to feel slightly more stress than their male fellow students, but those differences were not statistically significant, except on the requirement scale, which means that women were more burdened by requirements [21]. The author explains this effect with the fact that women deal more with their feelings and deal with them more freely and openly due to the influence of the culturally shaped gender role [21, 30] and, therefore, felt slightly more stressed.

However, we were able to find between-group differences for the QoL subscale social relationships, with women having higher scores than men. In a study by Leaune et al. [25], students reported poorer mental health and mental HRQoL, when they were affected by, for example, social isolation due to the lockdown in the COVID-19 pandemic or were female. In comparison, we might have found between-group differences. A possible explanation for that could be that most women provide and look more for social support than men [31]. Thus, they might have had the possibility of coping with stress by seeking help and social support [31]. This assumption is supported by a study by Abdullah et al. [32] who found higher levels of social interaction with family, friends and significant others to be predictors of higher psychological QoL. Shek [33] assumes that lower QoL in the social subscale could be explained by obligatory social isolation and, therefore, fewer social contacts and support during the COVID-19 pandemic. As social support is a protective factor against adversity, reduced social interaction is a threat.

Against our Hypothesis 3, there were no differences in scores of depressive symptoms for male compared to female participants. However, female participants reported higher anxiety scores than male participants, which could also be shown by Hinz & Schwarz [34]. This may be related to the higher prevalence for women compared to men for some anxiety disorders, for example, women are twice as likely as men to get generalized anxiety disorder [8]. Although it was not statistically significant, men tended to report higher depression scores than women. One possible explanation for this could be that men do not use social support as coping strategies as much as women do. Therefore, the higher social support potential of women can provide a “buffer” that can mitigate the effects of the contact restrictions on the risk of loneliness during the COVID-19 pandemic [35].

However, consistent with our first hypothesis, students without any anxiety and depression symptoms had higher QoL than students with such symptoms. This finding is also in line with Hansson [14], who found that people with major depressive disorder had lower subjective QoL than healthy subjects. We could not find an effect of gender on the four subscales of the WHOQoL-BREF (physical, psychological, social and environmental QoL). One explanation could be, that male and female participants had very similar life conditions in our study and, therefore, similar QoL. On the other hand, there was an effect of anxiety and depression on the four subscales of the WHOQoL-BREF, which is supported by Alsubaie et al. [19], who found that student participants without depression symptoms reported higher QoL scores in the psychological and social subscales of the WHOQoL-BREF. Considering anxiety, our findings are supported by Ghaedi et al. [20], who found significantly lower QoL in persons with social phobia than in a healthy control group.

In our study, the negative correlation between depression and the global QoL was higher than the correlation between anxiety and global QoL for both men and women, which is consistent with our second hypothesis and the findings of Hinz & Schwarz [34] who found that depression was more strongly associated with the total quality of life scale than anxiety. In the present study, correlations between global QoL and HADS raw depression scores were similar for men and women. Interestingly, men showed stronger correlations between global QoL and HADS raw anxiety scores, which means that men`s global QoL was worse with higher anxiety scores than women`s. This could be due to the fact that women are more open to psychotherapeutic treatments than men and, in addition to that, the majority of respondents in the study by Albani et al. [36] considered psychotherapy helpful in life crises and necessary for the successful treatment of certain mental disorders. This opinion is shared predominantly by women and less by men, which could indicate that men have a more stereotypical image of mental health issues and do not have the same courage as women to talk about their problems, which could have an impact on men`s QoL. Many studies found that anxiety and depression symptoms might lead to worse QoL [e.g. 9, 13, 14, 18]. Quilty et al. [2] could find a negative correlation of major depressive disorders with all three subscales of QoL (physical, emotional, social) of the Medical Outcome Study Health Survey, which is consistent with our findings. Social phobia correlated negatively with the emotional and social subscale [32], similarly, we found an effect of anxiety (and depression) on QoL subscales, including the psychological and social subscale.

To consider the possible role of the COVID-19 pandemic in relation to depression, anxiety and QoL, we compared our findings to previous literature. A study by Schmidt-Gürtler [21] found that 38% of included psychology students reported having clinically relevant depression scores. In our study, which took place during the COVID-19 pandemic period, many participants also studied psychology, but only 15.8% reported marginal to clinically significant depression scores, which is just slightly more than half as much as the author found before the pandemic. One possible explanation for this difference could be that our sample consists of students from different fields of study, compared to the sample of Schmidt-Gürtler [21], which consisted only of psychology students. In addition to that, the sample of Schmidt-Gürtler [21] was even smaller than our sample. Considering those numbers, it is imaginable, that psychology students, considered separately, experience more stress and worse QoL than a mixed sample of students with different study courses. Our findings considering the COVID-19 pandemic are partially consistent with findings from Aqeel et al. [37], who used the WHOQoL-BREF and found that there was an improvement of QoL from the first lockdown phase of the pandemic to the following phases, which could explain why some QoL means in several studies were higher during the pandemic than before. Maybe this is because people learned to adapt to this new situation and were able to handle it better. Similar to our study, Aqeel et al. [37] found that participants reporting moderate to severe levels of depression had lower levels of QoL compared to participants reporting normal to mild levels of depression. Further, Abdullah et al. [32] investigated QoL and associated factors among university students during the COVID-19 pandemic. They found lower QoL scores for the psychological and social subscales than in the non-pandemic general population. The perceived prevalence of COVID-19 in one’s own neighborhood and more severe depressive symptoms were associated with a poorer psychological QoL. In our study, we also found especially lower psychological and social QoL scores in students with depressive and anxious symptoms. Thus, the same subscales of QoL were affected in our participants and in the study of Abdullah et al. [32]. This may indicate that particularly social and psychological QoL were impaired by the COVID-19 pandemic. In a study by Wang et al. [38], 14% of students showed a moderate-to-severe level of depression and 38.48% showed a moderate-to-severe level of anxiety, which is very similar to our study in which about 16% reported marginal to clinically significant depression levels and about 29% reported marginal to clinically significant anxiety levels. Ratnani et al. [39] investigated the association between depression and social anxiety disorder and QoL. Here, it is possible to compare the means of the four subscales of the WHOQoL-BREF in participants with social anxiety and depression to our sample. Depression means were lower in our study for the psychological and social subscales, which could be explained by poor social interaction and support which may cause poorer psychological QoL. Considering the frequency of occurrence of anxiety and depression, it could be expected that anxiety and depression occurred more often during the pandemic. In the study by Ratnani et al. [39], 11.4% of participants (medical undergraduate students) suffered from clinically relevant social anxiety symptoms and 9% from clinically relevant depression symptoms. In our study, 11.4% of participants suffered also from anxiety (clinically significant level) and 5.6% from depression (clinically significant level). Anxiety scores were not different and depression frequency was even lower in our study compared to Ratnani et al. [39]. Comparing the means of the four subscales of the WHOQoL-BREF in participants with social anxiety and depression to our sample, the QoL means in the anxiety condition were lower in every subscale in our study during the pandemic compared to the study of Ratnani et al. [39], except for the physical subscale. QoL means in the depression condition were lower in our study for the psychological (*M* = 40.28) and social (*M* = 43.52) subscales compared to Ratnani et al. [39], *M*(psychological) = 54.00, *M*(social) = 59.29, which means that the participants of Ratnani et al. [39] were better socially integrated and had better psychological health in both conditions. This could be due to poor social interaction and help during the COVID-19 pandemic, which may cause poorer psychological and social QoL. However, we have to be careful comparing those values because, even though our sample size and the sample size of Ratnani et al. [39] are almost identical and both studies included students, they were both looking at two different samples.

Taken together our results show, that there are negative associations between the global, physical, psychological, social and environmental QoL and anxiety and depression. We did not find differences between men and women in three of the four QoL subscale scores, except for the social subscale. Moreover, we did not find differences between male and female participants for both of the global QoL items, either. Furthermore, our study revealed a significant main effect of anxiety and depression on physical, psychological, social and environmental QoL scores. Students’ QoL was highest if they were not affected by anxiety and depression. It was lowest in the psychological and social subscales if the students had marginal and especially clinically significant levels of anxiety and depression. Overall, our study has the strength of recruiting a large number of students coming from different fields of study. The sample included male and female students and individuals with mental health issues and there were no missing data.

Limitations on the other hand were, that while our study had a total sample of 297 participants, few of these actually had anxiety and depression symptoms (~ 15%). Moreover, one should consider that anxiety and depression are correlated and it often happens that they occur together, i.e. they are comorbid. Therefore, anxiety and depression are difficult to assess. In addition to that, we lost some of our potential participants as they did not complete the online survey at all. Perhaps more students would have taken part, if the survey had not taken place exclusively online. Furthermore, one must keep in mind that our sample consists of students from one German city only and therefore, it is not representative for the whole German population or students in other regions of Germany or other countries. Lastly, this study was a cross-sectional study.

## Conclusions

We cannot draw any conclusions about the direct consequences of the COVID-19 pandemic as our data were only collected at one time point during the pandemic and not before and/ or after. Differences in QoL may be due to anxiety and depression. However, people with poorer QoL may also be more susceptible to psychological distress and mental illness. We can conclude that participants without anxious and depressive symptoms have better physical, psychological, social and environmental QoL. If participants had significant anxiety or depression scores they had lower psychological and social QoL. The researched topic is highly relevant, especially considering the impact of the COVID-19 pandemic on mental health. Moreover, the focus on students enriches the current literature in this field and the inclusion of both male and female students adds value to the study by addressing gender differences in mental health issues. Although the findings indicate no significant differences in global QoL between genders, it highlights different impacts of anxiety and depression on QoL.

Future research should investigate a sample of participants with a larger percentage of people with mental health issues (especially anxiety and depression) in a longitudinal design to be able to draw conclusions about cause and effect. The review of Hohls et al. [40] summarizes longitudinal studies that deal with QoL of people with anxiety and depression. An expansion with the COVID-19 topic would be very interesting for future research. When designing such a study, one should consider studying known factors that improve QoL and are potentially modifiable clinical factors such as family psychoeducational programs, treatment of symptoms and better detection and evaluation [41]. Moreover, future research should have a larger sample size in more German or even international universities to allow generalizability. In addition, the role of exercising should be considered since this was found not only to improve QoL because it offers an opportunity for social interaction and goal-directed activity but also the symptoms of severe mental illnesses [42]. In addition to that, Harju & Bolen [43] found students with high optimism to have the highest overall QoL and satisfaction with their QoL. Pessimists reported lower overall QoL. This means that being generally optimistic may be a hint for better QoL, which could also be a part of future research.

### Electronic supplementary material

Below is the link to the electronic supplementary material.

**Additional file 1**: Tables A1 and A2

## Data Availability

The data are available from the corresponding author upon reasonable request.
