# Supplementary material for: Association between anxiety, depression and quality of life in male and female German students during the COVID-19 pandemic
Source: BMC Psychiatry. 2024 Mar 18;24:212. doi: 10.1186/s12888-024-05611-8 (PMC10949737; doi:10.1186/s12888-024-05611-8)
Supplement: Supplementary file 1 — Additional file 1: Tables A1 and A2 [file 12888_2024_5611_MOESM1_ESM.docx]

Additional Files

**Association between anxiety, depression and Quality of Life in male and female German students during the COVID-19 pandemic**

**Emily Wilzer, Annalena Zeisel, Veit Roessner, Melanie Ring***

*** Correspondence:** Melanie Ring, Department of Child and Adolescent Psychiatry, Faculty of Medicine Carl Gustav Carus, TUD Dresden University of Technology, Dresden, Germany. Email: [melanie.ring@ukdd.de](mailto:melanie.ring@ukdd.de)

**Table A1**

*Correlations of global Quality of Life (QoL) and Hospital Anxiety and Depression Scale (HADS) raw Depression and Anxiety scores separated by gender*

*Note*. ^**^The correlation is significant at the 0.01 level (2-sided). All presented correlations are Pearson-Correlations. ^a^Quality of Life. ^b^Hospital Anxiety and Depression Scale

|  | Global QoL^a^ | HADS^b^ Depression | HADS^b^ Anxiety |
| --- | --- | --- | --- |
| Male |  |  |  |
| Global QoL^a^ | 1 | -.47^**^ | -.38^**^ |
| HADS^b^ raw Depression | -.47^**^ | 1 | .51^**^ |
| HADS^b^ raw Anxiety | -.38^**^ | .51^**^ | 1 |
| Female |  |  |  |
| Global QoL^a^ | 1 | -.40^**^ | -.22^**^ |
| HADS^b^ raw Depression | -.40^**^ | 1 | .30^**^ |
| HADS^b^ raw Anxiety | -.22^**^ | .30^**^ | 1 |

**Table A2**

*Overview of the answers to the global items of the World Health Organization Quality of Life Brief Version (WHOQoL-BREF)*

|  | Male | | Female | |
| --- | --- | --- | --- | --- |
|  | N | % | N | % |
| Global QoL^a^ ^b^ |  |  |  |  |
| Very bad | 1 | 0.8 | - | - |
| Bad | 4 | 3.3 | 7 | 4 |
| Medium | 23 | 19 | 32 | 18.2 |
| Good | 79 | 65.3 | 106 | 60.2 |
| Very good | 14 | 11.6 | 31 | 17.6 |
| Global satisfaction with health^c^ |  |  |  |  |
| Very dissatisfied | 3 | 2.5 | 4 | 2.3 |
| Dissatisfied | 10 | 8.3 | 13 | 7.4 |
| Neither satisfied nor dissatisfied | 21 | 17.4 | 37 | 21 |
| Satisfied | 63 | 52.1 | 92 | 52.3 |
| Very satisfied | 24 | 19.8 | 30 | 17 |

*Note*. ^a^Quality of Life ^b^Global Item 1 of the World Health Organization Quality of Life Brief Version (WHOQoL-BREF) ^c^Global Item 2 of the WHOQoL-BREF
